# Anatomical 3D‐printed metaphyseal cone in revision total knee arthroplasty: A short‐term clinical and radiological follow‐up evaluation

**DOI:** 10.1002/jeo2.70754

**Published:** 2026-06-14

**Authors:** Federica Rosso, Vincenzo Mattiacci, Matteo Bruzzone, Federico Dettoni, Umberto Cottino, Roberto Rossi

**Affiliations:** ^1^ Department of Orthopaedics and Traumatology, AO Ordine Mauriziano University of Torino Torino Italy; ^2^ Department of Orthopaedics and Traumatology Università degli Studi di Torino Torino Italy; ^3^ Department of Orthopaedics and Traumatology Ospedale Cardinal Massaia Asti Italy

**Keywords:** arthroplasty, cones, knee, metaphyseal, revision

## Abstract

**Purpose:**

Bone loss is commonly encountered during revision total knee arthroplasty (R‐TKA), and metaphyseal cones are often used to address these defects. In addition, they can enhance Zone 2 fixation in R‐TKA in the presence of poor bone quality. The aim of this study was to evaluate the short‐term clinical and radiological outcomes of a novel three‐dimensional (3D)‐printed metaphyseal cone in R‐TKA.

**Methods:**

This single‐centre, single‐surgeon prospective study included all patients who underwent R‐TKA using a novel 3D‐printed anatomical metaphyseal cone (AMF, ENOVIS®) with a minimum follow‐up of 24 months. Patient demographics and operative details were recorded. Postoperative assessments included the Knee Society Score (KSS), Oxford Knee Score (OKS) and Short Form‐12 (SF‐12). Radiographic evaluation focused on radiolucent lines, implant or cone loosening and the cone's ability to replicate proximal tibial anatomy. Complications, reoperations and revisions for any reason were documented.

**Results:**

Thirty patients (31 knees) underwent R‐TKA with a total of 48 cones (24 tibial and 24 femoral), with a mean follow‐up of 37.3 months (standard deviation [SD] 8.8). In 48.4% of cases, cones were used on both the tibial and femoral sides. No intraoperative fractures occurred during cone preparation. All patients demonstrated significant improvement in clinical outcomes. Radiographic evaluation showed no evidence of cone loosening and accurate reproduction of proximal tibial anatomy. Survivorship free from cone revision, including for aseptic loosening, was 100%. Survivorship free from any revision or reoperation was also 100%.

**Conclusion:**

This novel 3D‐printed anatomical metaphyseal cone demonstrated excellent short‐term outcomes, with no early loosening and accurate replication of proximal tibial morphology. However, longer‐term follow‐up and a larger cohort are required to confirm these preliminary findings.

**Level of Evidence:**

Level IV.

**Abbreviations**
AORI: Anderson Orthopedic Research Institute
AP: anteroposterior
CT: computer tomography
KSS: Knee Society Score
LL: latero‐lateral
OKS: Oxford Knee Score
PJI: periprosthetic joint infection
RHK: rotating hinge knee
ROM: range of motion
R‐TKA: revision total knee arthroplasty
SD: standard deviation
SF‐12: Short Form‐12
TKA: total knee arthroplasty
VVC: varus valgus constrained

## INTRODUCTION

The number of revision total knee arthroplasties (R‐TKAs) is projected to rise in the coming years, primarily due to the increasing number of primary total knee arthroplasty (TKA) procedures and a decreasing average patient age [20]. Some projections estimate that the number of R‐TKAs will increase by 189% by 2030 [38].

Infection remains one of the leading causes for TKA failure, followed by instability and aseptic loosening [15]. Regardless of the cause of failure, surgeons may encounter several challenges during revision TKA, including bone loss and poor bone quality. Bone loss has historically been classified using the Anderson Orthopedic Research Institute (AORI) classification [16] and different treatment options have been proposed, including metaphyseal cones, which have shown good outcomes [29, 30, 37]. Furthermore, the crucial role of bone quality in achieving proper implant fixation and ensuring long‐term survivorship has been clearly demonstrated. In 2015, Morgan‐Jones introduced the concept of ‘zonal fixation’, emphasizing the importance of adequate metaphyseal fixation, either with cones or sleeves [29]. Many authors consider metaphyseal cones or sleeves the best option to fill metaphyseal bone defects, particularly due to their availability and simplicity compared to techniques such as bone impaction grafting or structural allografts [30]. However, concerns have been raised about their higher costs and the need to sacrifice viable bone during implantation, especially with first‐generation metaphyseal cones [37]. To overcome these limitations, new ‘anatomical’ cones have been developed to improve fixation, reduce bone sacrifice and better replicate distal femoral and proximal tibial anatomy. The aim of this study was to describe the short‐term clinical and radiological outcomes of a new generation of three‐dimensional (3D)‐printed anatomical metaphyseal cones designed both as a filler and to enhance metaphyseal fixation.

## METHODS

### Patients' demographics and evaluation

This prospective study evaluated a consecutive series of R‐TKAs performed at a single institution (AO Ordine Mauriziano) by the same surgeon (R. R.) using a second‐generation 3D‐printed anatomical metaphyseal cone (AMF, ENOVIS®) between May 2021 and July 2023.

Inclusion criteria were R‐TKAs performed using the AMF® cones (ENOVIS®) for any reasons with a varus valgus constrained (VVC) or rotating hinge knee (RHK) implant, with minimum of 2 years of follow‐up and complete preoperative and postoperative clinical and radiological evaluation. Exclusion criteria included the use of cones in primary TKA or in combination with any mega‐prosthesis. All patients underwent a standardized preoperative workup to identify the cause of failure, including assessment for periprosthetic joint infection (PJI), a complete series of X‐rays and computed tomography (CT) scans to evaluate bone loss [11].

Standard clinical evaluation focused on tibiofemoral stability, patellofemoral tracking and ROM. The cause of failure was classified according to Vince et al. into nine categories: (1) aseptic loosening, (2) instability, (3) patellar com‐ plications and malrotation, (4) structural failure of the implant, (5) PJI, (6) extensor mechanism rupture, (7) stiffness, (8) periprosthetic fracture and (9) no diagnosis, the so‐called mystery knee [40]. Patients' demographics are summarized in Table 1.

**Table 1:** Demographic characteristics of included patients.

	Overall (*n* = 30)
Male no. (%)	11 (36.7)
Age (years)	72.4 ± 10.1
BMI (kg/m^2^)	28 ± 4.6
Cause of revision no. (%)	
Aseptic loosening	24 (77.4)
Infection	3 (9.7)
Malpositioning	4 (12.9)
Implant constraint no. (%)	
CCK	25 (80.6)
PS	5 (16.1)
RHK	1 (3.4)
Stems no. (%)	
TibiaLong	5 (16.1)
Short	24 (77.4)
FemurLong	2 (6.5)
Short	29 (93.5)
AORI classification no. (%)	
Femur	
1	14 (45.2)
2A	6 (19.4)
2B	10 (32.3)
3	1 (3.2)
Tibia	
1	15 (48.4)
2A	10 (32.3)
2B	5 (16.1)
3	1 (3.2)
Follow‐up (months)	37.3 ± 8.8

Abbreviations: AORI, Anderson Orthopedic Research Institute; BMI, body mass index; CCK, constrained condylar knee; kg, kilograms; m, metre; no. number; PS, posterior cruciate substituting; RHK, rotating hinge knee.

### Surgery‐related data

All surgical procedures were performed by a single experienced surgeon (R. R.) using a standardized approach. The choice of implant, level of constraint, use of stems and soft‐tissue balancing techniques were tailored according to the patient's specific pathology and intraoperative findings. A semi‐constrained or rotating‐hinged implant was used depending on the extent of bone loss and ligamentous deficiency. The senior author's indications for a rotating‐hinged implant included: (1) disruption of both collateral ligaments or one collateral ligament with posterior capsule disruption, (2) uncorrectable flexion/extension mismatch, (3) dislocation, (4) extensor mechanism insufficiency, (5) neuromuscular condition and (6) revision of a previous rotating‐hinged implant [34].

In case of prosthetic joint infection, a two‐stage revision was performed using mobile or static cement spacers, depending on the presence of severe ligamentous instability, insufficient extensor mechanism, massive bone loss or compromised soft tissue. The second stage of surgery was performed after laboratory tests and clinical exams were negative for infection [28].

All R‐TKAs were performed according to the ‘three‐step technique’ described by Kelly Vince [41]. The tourniquet was inflated only during cementation. All the implants were cemented with antibiotic‐loaded cement.

Stems were used in all cases: long stems for strong diaphyseal fixation and offset stems if one of the following conditions was present: (1) need for malalignment correction, (2) anatomical mismatch between the metaphysis and diaphysis centres or (3) need to improve gap balancing [3]. In other cases, short‐cemented stems were preferred.

Bone loss and quality were classified using the modified AORI classification previously published by the Authors (Table 2). ‘Good bone quality’ was mostly observed in revision for instability, patellofemoral conditions or malalignment. ‘Sclerotic (S)’ bone quality was defined as insufficient bleeding after bone preparation, lack of trabecular structure and the characteristic ‘marble appearance’, and it was commonly seen after antibiotic spacers, in case of multiple revisions or in severe aseptic loosening. ‘Osteoporotic (O)’ bone quality was defined as good bleeding is present after bone preparation, but with increased pore size in the trabecular structure, resulting in suboptimal bone quality. The distinction between good bone quality, sclerotic and osteoporotic bone can help the surgeons in choosing or not to use a metaphysis augment [34].

**Table 2:** The modified AORI classification from a previous study [3].

AORI	Bone quality	Treatment option
F1‐T1	Good (G)	– <5 mm (<50% of bone surface area): Cement and morselized bone – 5–10 mm: Cement and screw or morselized bone
	Sclerotic (S) or osteoporotic (O)	– Ensure adequate Zone 3 (diaphysis) fixation – If very sclerotic bone: Consider small tantalum cone (disadvantage: sacrifices bone stock)
F2A‐T2A	Good (G)	– 5–10 mm: Cement and screw (only for low‐demand patients) – >5 mm (>40% of surface unsupported from host bone): Metal augments or structural allograft/impaction bone grafting (young patients) – Ensure adequate zone 3 fixation (stems)
	Sclerotic (S) or osteoporotic (O)	Same as above – Adequate Zone 2 fixation (cone) is strongly recommended to reduce the risk of aseptic loosening
F2B‐T2B or Type 3 defect	Good (G)	– Impaction bone grafting (young patients), metal augments, structural allograft – Larger defect: Tantalum cone and titanium sleeve with short‐ to medium‐length stems – Severe Type 3 defect: Mega prosthesis
	Sclerotic (S) or osteoporotic (O)	Same as above – Tantalum cones are often required to treat bone loss and enhance Zone 2 fixation

Abbreviations: A, unicondylar involvement; AORI, Anderson Orthopedic Research Institute; B, bicondylar involvement; F, femoral; T, tibial.

Metaphyseal cones were indicated in three main scenarios: (1) bone loss requiring filling (AORI 2B or 3) [16]; (2) small defect with poor bone quality in association with diaphyseal stems to achieve at least two valid fixation zones and (3) small bone defects with short cemented stems to achieve adequate fixation in Zones 1 and 2 [12, 35].

### Characteristics of the metaphyseal cones

The 3D‐printed anatomical metaphyseal cones used in this study (AMF, ENOVIS®) are made of porous titanium, with 65% of porosity and an average pore diameter of 640 µm. Their elasticity closely mimics natural bone, enabling even load distribution [26, 27] and their high osteointegration properties have already been demonstrated. In vitro studies proved that mineralized extracellular matrix deposition, proliferation and differentiation of osteoblasts occur regardless of osteogenic factors [8, 9, 18].

These findings translate into effective osseointegration in vivo, with ingrowth into trabecular and cortical bone, increasing bone formation compared to traditional coatings [14].

The tibial cone is available in the central or peripheral configuration. The central configuration has a conic shape with posterior inclination, replicating the posterior proximal tibial cortex. Similarly, the conic configuration in the anteroposterior (AP) view again reproduces the conical morphology of the proximal tibia.

**Figure 1:**
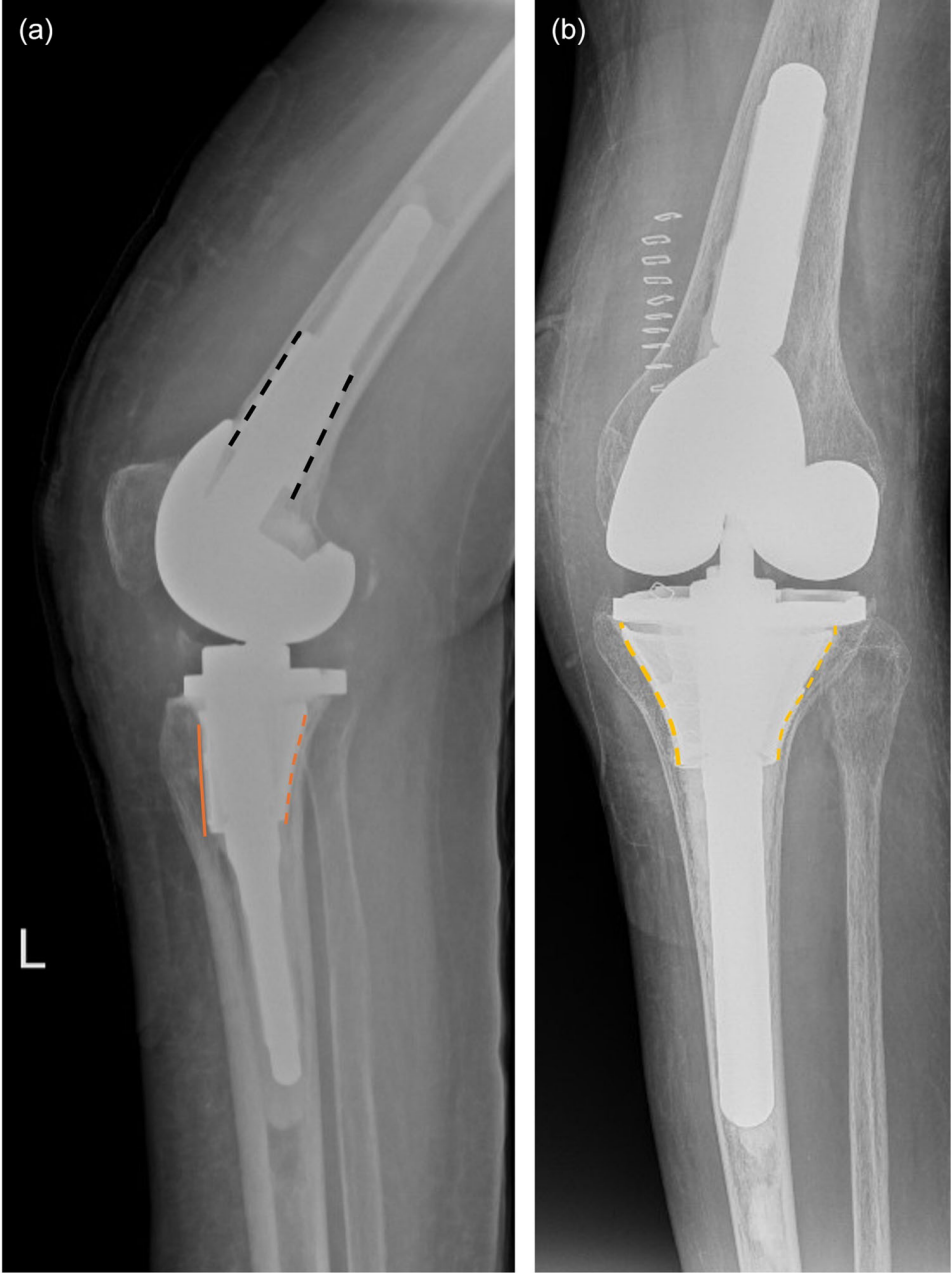
X‐ray lateral (a) and antero‐posterior (b) view demonstrating the shape of a femoral and tibial central cone. Black dotted line underlines the congruency between cone and bone shape. Orange dotted line underlines the same congruency on the tibial side.

The femoral cone is available in a central configuration and a bicondylar one, with the latter featuring an asymmetric condylar design for left and right sides. Bone preparation is performed with dedicated instrumentation, including reamers instead of broaches, to reduce the risk of intraoperative fractures [7, 39]. Figure 1 shows x‐rays of two types of tibial cones.

### Clinical and radiological evaluation

The Knee Society Score (KSS), Oxford Knee Score (OKS) [21] and the Short Form‐12 (SF‐12) [22], as well as a complete clinical evaluation including alignment, knee stability and range of motion (ROM), were used to assess pre‐ and postoperative outcomes at 2 months, 1 year and annually thereafter. All evaluations were performed by a third orthopaedic surgeon not involved in the surgery.

All patients underwent pre‐ and postoperative complete series of x‐rays at 2 months, 1 year, and therefore annually, including a long‐leg view of the lower limb. Overall limb alignment, as well as component positioning and presence of a radiolucent line (progressive or not), were evaluated according to the Knee Society Roentgenographic Evaluation System [17].

**Figure 2:**
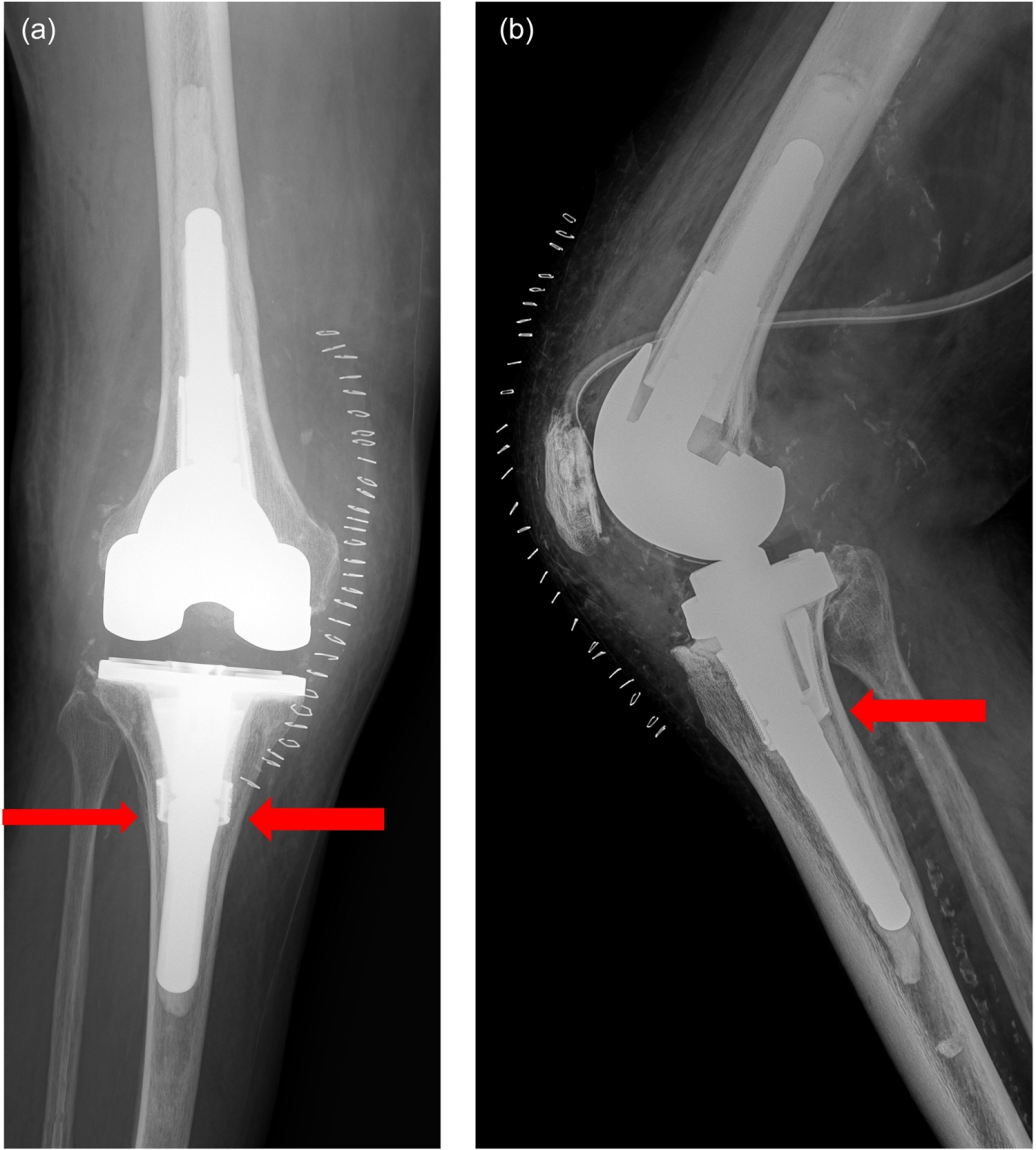
X‐ray antero‐posterior (a) and lateral (b) view demonstrating the area where cone‐cortical bone impingement may occur (red arrows).

**Figure 3:**
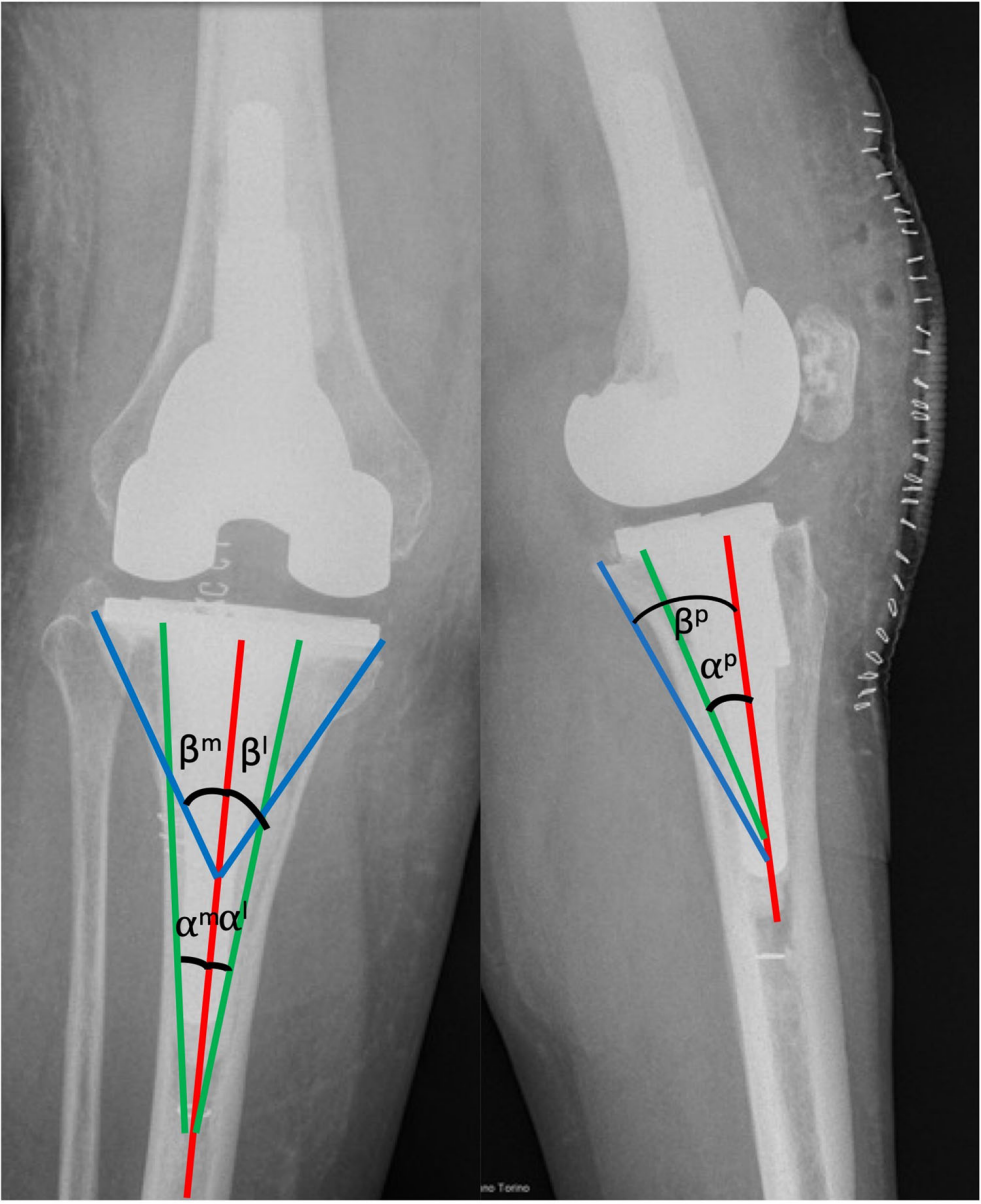
X‐rays demonstrating the evaluation of anatomy reproduction using the ratio between cone inclination (*α* ^m,l,p^) and tibial inclination (*β* ^m,l,p^). Red line: tibial anatomical axis; green line: tangent to the cone; blue line: tangent to the cortical bone. *α* ^m^ = medial cone inclination; *α* ^l^ = lateral cone inclination; *α* ^p^ = posterior cone inclination; *β* ^m^ = medial tibial inclination; *β* ^l^ = lateral tibial inclination; *β* ^p^ = posterior tibial inclination.

Cone position relative to cortical bone (impingement) was evaluated on both anteroposterior (AP, medial and lateral impingement) and lateral (latero‐lateral [LL], posterior impingement) x‐rays on the tibial side (Figure 2). The ability of the cone to reproduce proximal tibial anatomy was assessed by calculating the ratio of the tangent to the cone and the tangent to the tibia (both based on the tibial axis). This ratio was chosen to minimize issues related to x‐ray acquisition (Figure 3). On the femoral side, the same measurements were not possible due to implant overlap.

### Data analysis

Descriptive statistics were used for all demographic, subjective and objective results. Data were collected using Excel® (Microsoft®) and presented as mean, standard deviation (SD) and range. *T* tests and *χ* ^2^ tests were used to analyse differences in continuous and categorical variables, respectively. The Kaplan–Meier method was used to evaluate cumulative survivorship in the presence of failures.

### The institutional review board (IRB) approval

The research is conducted in alignment with the ethical principles outlined in the 1964 Declaration of Helsinki as well as the Health Insurance Portability and Accountability Act (HIPAA) guidelines. The IRB at the author's institution classified this research as exempt from IRB oversight (a prospective study on an established surgical method). Oral informed consent for participation in the study was secured from each patient included.

## RESULTS

Thirty patients (31 knees) met the inclusion criteria and were enroled in the study. Those patients underwent R‐TKA with 48 cones (24 tibial and 24 femoral) with an average follow‐up of 37.3 months (SD 8.8; range: 24–54.8). All the surgeries were performed by the senior author (R. R.). The mean age at surgery was 71.5 years (SD 10.1; range: 48.5–84.4), with 63.3% female and an average BMI of 28 kg/m^2^ (SD 4.6; range: 21.1–38.5).

The predominant indication for revision was aseptic loosening (24 cases, 77.4%), and in most of the cases (80.6% of patients) a condylar constrained implant was used. Detailed demographic and clinical characteristics are summarized in Table 1.

Bone losses were classified according to the AORI classification, with most of defects classified as AORI 1 (45.2%), AORI 2A (19.4%), AORI 2B (32.3%) and AORI 3 (3.2%) (Table 2). In these cases, the cone was used to achieve a Zone 2 fixation due to poor epiphyseal bone quality, in association with a short‐cemented stem.

In 16 patients (48.4%), both femoral and tibial cones were implanted. Twenty‐four tibial cones were used, mainly the 18 mm central cone (14 cones, 58.3%). Twenty‐four femoral cones were used, mostly an 18 mm central cone (13 cones, 56.5%) with only one bicondylar femoral cone. All cones are summarized in Table 3.

**Table 3:** Characteristics of included cones.

Cones no. (%)	
Tibia	24
15 mm	2 (8.3)
18 mm	14 (58.3)
21 mm	8 (33.3)
Femur	24
15 mm	6 (25)
18 mm	13 (54.2)
21 mm	5 (20.8)
Cones distribution (%)	Overall (*n* = 31)
Tibial only	25.8
Femur only	25.8
Both tibia and femur	48.4

Stems were used in all cases, mostly short (<100 mm) cemented stems on both femoral and tibial sides (77.4% and 93.5% respectively, Table 1). No intraoperative fractures occurred during cone preparation.

At the final follow‐up, all patients demonstrated significant (*p* < 0.005; Table 4) improvement in clinical outcomes compared to preoperative values. Particularly, KSS increased from a preoperative mean of 44.5–67.7 at final follow‐up. Similarly, OKS improved from 22.5 before surgery to 35.3 at the last evaluation. Finally, the SF‐12 score rose from 25.6 preoperatively to 40 at final follow‐up.

**Table 4:** The preoperative and postoperative KSS, OKS and SF‐12 scores and their *p* value.

	Preoperative				Postoperative						*p* value
	Mean	Median	Interquartile range	SD	Range	Mean	Median	Interquartile range	SD	Range	
KSS	44.5	43	33.5–57	19	13–97	67.7	66.7	56.2–85	20.3	30.7–100	*p* < 0.001
OKS	22.7	20	15.5–31.5	9.7	6–40	35.3	33.0	25.9–46	15.5	8–80	*p* < 0.001
SF‐12	25.6	25.0	23.3–28.0	5.1	15–35	40	40	37.0–43.0	5.9	30–50	*p* < 0.001

Abbreviations: KSS, Knee Society Score; OKS, Oxford Knee Score; SF‐12, Short Form‐12; SD, standard deviation.

Radiographic evaluation demonstrated that no progressive radiolucent lines greater than 2 mm were observed at the bone–cone or bone–implant interfaces. There was no evidence of cone or implant loosening, migration or subsidence in any case. In 19.4% of cases, some non‐progressive radiolucent line was detected, particularly on the tibial side, representing probably some are of bone sclerosis. Representative radiographs are shown in Figures 1 and 2.

The metaphyseal cones demonstrated good anatomical fit of the cones, with accurate replication of the proximal tibial anatomy, with small SD of the cone‐metaphyseal bone fill ratio values. The ratio was 1.9 (SD 0.4; range: 1.18–2.7) for the medial AP inclination, 1.8 (SD 0.3; range: 1–2.3) for the lateral AP inclination, and it was 1.4 (SD 0.1; range: 1–1.7) for the posterior LL inclination.

No cases of early or late infection, periprosthetic fracture, extensor mechanism disruption or wound healing complications were observed during the follow‐up period. There were no reoperations or revisions involving the metaphyseal cones or the revision prostheses. The Kaplan–Meier estimated survivorship free from cone revision for any reason was 100% at the latest follow‐up. Survivorship free from any revision or reoperation was also 100%.

## DISCUSSION

This prospective study demonstrates that the use of a novel, 3D‐printed anatomical metaphyseal cone in 31 revision TKA provides good short‐term clinical and radiological outcomes. The cones showed reliable metaphyseal fixation, accurate anatomical fit and favourable osseointegration, with no cases of loosening, migration or failure during a mean follow‐up of 37.3 months. These results are consistent with previously published reports on other metaphyseal cones [4, 13, 32].

Severe tibial metaphyseal bone loss remains a persistent challenge in revision TKA, and several strategies to address bone losses have been described [12, 17, 19], including structural allografts, which have played a major role for decades. However, structural allografts have been progressively abandoned due to long operating times, limited availability of suitable grafts, nonunion, delayed union and graft resorption, with a ten‐year revision rate exceeding 20% in some series [2, 5, 10].

Porous metal cones and sleeves have gained popularity due to low complication rates and excellent osteointegration [3, 6, 34, 41]. Furthermore, in 2015, Morgan‐Jones introduced the concept of ‘zonal fixation’, highlighting the importance of achieving fixation in at least two out of the three available zones in revision TKA and emphasizing the crucial role of the metaphysis [23]. Given the osteoconductive nature of porous titanium, cones have been proposed to enhance fixation in the metaphyseal region, reducing stress on the epiphyseal bone and lowering the risk of aseptic loosening [29, 32]. In the last 10 years, metaphyseal cones have therefore been used in three main clinical scenarios: (1) severe defects as structural fillers, (2) moderate defects with poor bone quality combined with long diaphyseal‐engaging stems and (3) moderate or small defects with preserved bone quality using short‐cemented stems. However, first‐generation metaphyseal cones were mainly designed as defect fillers in sclerotic bone and were often bulky, with small internal diameters and broach‐based preparation. This could limit stem diameter, prevent the use of offsets or result in suboptimal canal fill ratio (CFR) [36]. On the tibial side, even the smallest cones could occasionally be too large, risking impingement on the medial, lateral or posterior cortex. To address the limitations of first‐generation cones, including the significant amount of bone removal required even for the smallest cone, new solutions have been proposed, such as customized 3D‐printed metaphyseal cones with good outcomes [31], although their high‐cost limits routine use [31, 33]. The anatomical cones evaluated in this study address these concerns by closely matching native bone geometry and reducing the need for additional bone resection, without further costs compared to first‐generation cones.

Their anatomical shape, characterized by a more posterior inclination and a conical tibial profile, enabled accurate metaphyseal reproduction. Conicity ratios between the tibial bone and the cone ranged between 1.5 and 1.9 with minimal variance, confirming a good fit, with few cases of impingement in very small patients.

Furthermore, no intraoperative fractures were encountered, even in cases of severe sclerotic bones, possibly due to the reamer‐based preparation system [13]. Even if the follow up if short, there were no progressive radiolucent lines or signs of cone loosening, as well as no major complications.

These findings are consistent with previous reports on metaphyseal cones, which have shown high rates of implant survivorship, low rates of aseptic loosening and favourable clinical outcomes [25, 30, 33]. Olhmeier et al., using calcium phosphate‐coated porous tibial cones, demonstrated reliable fixation: among 52 cones, only four revisions occurred (7.7%), and 78.6% of radiographs showed stable integration with minimal radiolucency [31]. Tetrault et al., in a larger sample of 202 anatomical cones, reported a survivorship free of cone revision for aseptic loosening of 100% and survivorship free of any cone revision of 98%. Survivorships free of any revision and any reoperation were 90% and 83%, respectively [39].

The results of this study, with a great number of short stems associated with the metaphyseal cone, underlined one more advantage of the metaphyseal fixation. Reducing the length of stems may reduce the stress shielding and/or toggling at the interface between the host bone and the tibial component, contributing to reducing the rate of end‐of‐stem pain [24].

Furthermore, recent studies have shown that tibial short stems, when combined with trabecular cones, perform as well as long stems. In a retrospective, multi‐centre study, Batinica et al. reported favourable results in terms of radiolucencies and failure‐free survival for both short and long stems, with no statistically significant differences between the two groups [1].

This study has limitations. The sample size is relatively small, and the follow‐up period is limited to short‐term outcomes, which may not be enough to evaluate cone loosening. However, this limitation is strictly related to the recent introduction of these cones on the market. The absence of a control group using traditional cones limits the ability to draw direct comparative conclusions. Assessment of anatomic reproduction was based solely on radiographs, and evaluation of femoral cones was limited by implant overlap. Although the tibial side, where fixation is often more challenging, could be reliably assessed radiographically, CT‐based 3D analysis would provide a more comprehensive evaluation of bone filling and potential impingement.

## CONCLUSION

This prospective study demonstrates that anatomically shaped 3D‐printed metaphyseal cones prepared with a reamer‐based system provide good early fixation and accurate metaphyseal reconstruction in revision TKA. Their anatomical profile, low rate of cortical impingement and easy reamer‐based preparation are a meaningful evolution from first‐generation designs. Future studies with larger patient cohorts, longer follow‐up and randomized designs are warranted to confirm these encouraging findings.

## AUTHOR CONTRIBUTIONS

**Federica Rosso**: Conceptualization; writing; review and editing. **Vincenzo Mattiacci**: Data curation; writing. **Matteo Bruzzone**: Supervision; validation. **Federico Dettoni**: Visualization; investigation. **Umberto Cottino**: Investigation; validation. **Roberto Rossi**: Conceptualization; supervision; review and editing.

## CONFLICT OF INTEREST STATEMENT

Federica Rosso: Research grant from Medacta, Committee member, Siagascot, European Knee Society. Matteo Bruzzone: Teaching consultant, DePuy. Federico Dettoni: Board member OTODI. Umberto Cottino: Teaching consultant, Zimmer Biomet. Roberto Rossi: Angelini Farmaceutica: Paid presenter or speaker; Arthrex, Inc: Paid presenter or speaker; DePuy, A Johnson & Johnson Company: Paid presenter or speaker, Lima corporate: IP royalties Zimmer: Paid consultant; Paid presenter or speaker. Committee Member, Siagascot, European Knee Society, American Knee Society, AAHKS international member, Deputy Editor, JAAOS Global Research & Reviews. The remaining author declares no conflict of interest.

## ETHICS STATEMENT

The research is conducted in alignment with the ethical principles outlined in the 1964 Declaration of Helsinki as well as the HIPAA guidelines. The Institutional Review Board (IRB) at the author's institution classified this research as exempt from IRB oversight (a prospective study on an established surgical method).

## Data Availability

Oral informed consent for participation in the study was secured from each patient included.
